# Editorial: Neuro-detection: advancements in pattern detection and segmentation techniques in neuroscience

**DOI:** 10.3389/fncom.2025.1685174

**Published:** 2025-09-02

**Authors:** Najib Ben Aoun, Sadique Ahmad, Ridha Ejbali

**Affiliations:** ^1^Faculty of Computing and Information, Al-Baha University, Al Baha, Saudi Arabia; ^2^EIAS: Data Science and Blockchain Laboratory, College of Computer and Information Sciences, Prince Sultan University, Riyadh, Saudi Arabia; ^3^Research Team in Intelligent Machines (RTIM), Engineering School of Gabès (ENIG), University of Gabès, Gabès, Tunisia

**Keywords:** neuroscience, artificial intelligence, image segmentation–deep learning, brain tumors classification, neuroimaging, medical imaging

In recent years, the intersection of artificial intelligence (AI), deep learning, and neuroimaging has created transformative opportunities in the early diagnosis, classification, and treatment planning of neurological disorders and brain tumors. This Research Topic, *Neuro-detection: Advancements in Pattern Detection and Segmentation Techniques in Neuroscience*, presents a collection of nine high-quality studies that illustrate the growing impact of machine learning-driven methods in advancing medical diagnostics and understanding complex neurobiological conditions. The collection explores how AI techniques, particularly deep neural networks, explainable AI (XAI), unsupervised learning, and ensemble models, can be effectively applied to tasks such as interpreting complex medical imaging data, uncovering subtle biomarkers, and supporting clinical decision-making. Each contribution not only advances methodological concepts but also demonstrates the applicability and generalizability of these techniques in real-world diagnostic scenarios.

Brain tumor classification and segmentation remains a particularly challenging problem due to tumor heterogeneity, imaging variability, and anatomical differences across patients. Several contributions address these challenges with state-of-the-art approaches. One study proposes an ensemble model combining Vision Transformers (ViT) and EfficientNet-V2, optimized via a genetic algorithm-based weighted strategy. This hybrid system captures both local and global MRI features, outperforming individual models with 95% classification accuracy. In this contribution, Gasmi et al., demonstrate that such synergistic model integration can address multi-class medical classification problems with potential applicability beyond neuro-oncology.

Kiran et al., introduce a binary convolutional neural network (BCNN) for segmenting the ten most common brain tumor types, supported by a new dataset of 6,600 MRI images. Using adaptive thresholding and advanced morphological operations, the BCNN achieved 99.40% accuracy and a 99.28% F1-score, excelling at differentiating tumor grades and types. Another contribution, by Albalawi et al., presents a multi-task CNN architecture capable of simultaneously detecting tumors, classifying them by type and grade, and localizing them. Trained on over 7,000 MRI images across four categories, the model achieved 99% overall accuracy, making it a promising candidate for integration into clinical workflows.

Beyond supervised segmentation, Arora et al., propose an unsupervised “unruly clustering” method based on integrated intuitionistic fuzzy logic with conditional spatial properties. By incorporating hesitation degrees and conditional spatial functions, the approach adapts to local image context, improving segmentation robustness in noisy or low-contrast conditions. Similarly, Iqbal et al., bridge handcrafted statistical radiomic features with deep spatial representations from a ResNet-inspired architecture. Their custom Fusion Net preserves critical information from both domains, reaching 97.53% accuracy and 97.77% precision on the Brains dataset, underscoring the benefits of combining engineered and learned features for complex classification tasks.

Moreover, AI applications in neurodegenerative disease diagnosis extend these innovations beyond oncology to conditions like Alzheimer's disease (AD) and Parkinson's disease (PD), where early detection can substantially influence patient outcomes. In AD, graph-based modeling has emerged as a powerful paradigm for capturing the complex interplay between brain regions. Alharbi et al., introduce a spectral graph Convolutional Neural Network (SGCNN) that represents MRI-derived brain connectivity as a graph, enabling the model to learn topological and spectral patterns linked to disease progression. Through targeted ablation experiments, they improved classification performance to 95%, outperforming conventional CNNs and confirming that graph-based architectures are well suited for mapping the connectivity disruptions and structural atrophy patterns characteristic of AD.

Building on this connectivity focus, Biswas and Sripada shift from *associative* to *causal* brain network analysis, introducing Causal Functional Connectivity (CFC) as a richer and potentially more prognostic biomarker. Using resting-state fMRI data from the Alzheimer's Disease Neuroimaging Initiative, they applied the Time-aware PC (TPC) algorithm—a directed graphical modeling method tailored for time series—to compute whole-brain causal connectomes for cognitively normal, mild cognitive impairment, and AD groups. Compared to Granger Causality and Sparse Partial Correlation approaches, TPC produced sparse, interpretable, and directionally meaningful maps that aligned with known connectivity patterns while revealing contemporaneous and directional influences often missed by standard methods. Edge-wise statistical analysis highlighted reduced causal influence from Heschl's gyrus, thalamus, and posterior cingulate cortex, alongside increased self-connections in the parahippocampal gyrus—alterations consistent with more than 30 prior neuroimaging studies. These findings capture both the loss of key network hubs and the emergence of compensatory hyperconnectivity during early disease stages, positioning TPC-based CFC analysis as a valuable complement to graph-based classifiers like SGCNN in early AD detection.

While AD studies highlight the role of connectivity modeling, PD research in this Research Topic demonstrates the potential of alternative, non-imaging biomarkers. Kim et al., present a voice-based diagnostic framework using self-supervised deep representation pattern learning (SS-DRPL), which learns micro-temporal and frequency features from unlabelled voice recordings. Combined with LSTM-RNN and DNN architectures, the model achieved an F1-score of 0.94, showing the promise of accessible, non-invasive diagnostic tools.

Another PD-focused study by Alharthi applies XAI techniques, particularly layer-wise relevance propagation (LRP), to neural network models trained on gait sensor data from Parkinson's and cognitively impaired individuals. The approach not only achieved a 98% F1-score on PD datasets and up to 90% ± 10% on healthy individuals under dual-task conditions, but also provided interpretable insights into how specific gait features influence predictions. Such transparency strengthens clinician trust and enables better understanding of how neurological deterioration manifests in motor patterns.

Together, these contributions present a coherent picture of progress at the intersection of deep learning, neuroimaging, and medical diagnostics. Across both tumor detection and neurodegenerative disease diagnosis, common themes emerge: integrating multi-modal and hybrid feature sets strengthens model performance; graph-based and causal network modeling open new frontiers for understanding disease mechanisms; and XAI techniques enhance transparency, paving the way for clinical adoption. The diversity of datasets, modalities, and analytic strategies showcased here demonstrates both the adaptability of AI methods to different neurological conditions and their potential to transform diagnostic practice in the years ahead.

